# Surgical Innovation, a Niche and a Need

**Published:** 2012

**Authors:** Fatemeh Heidary, Reza Gharebaghi

**Affiliations:** 1 School of Medicine, Shahid Beheshti University of Medical Sciences, Tehran, Iran; 2 Editorial Office, Medical Hypothesis, Discovery & Innovation Ophthalmology Journal

**Keywords:** Ophthalmology, Hypotheses, Surgical innovation, techniques, Idea

**Surgical Innovation** **in the current**

The life sciences in the 21st century are evolving from a descriptive or phenomenological discipline to extrapolative one. Rapid development has transpired dramatically in biological studies in the last few years, much of which encompasses concepts more familiar to engineers and physicists rather than physicians [1]. 

Innovation has vast morphological meanings but is simply defined as the act of announcing something new or the use of a novel idea or technique. In some cases, it is practiced as replacement to invention, though innovation is further accurately outlined as something thought up or mentally fabricated. Although innovation in surgery has a less than perfect tradition, the field and research of surgical innovation are new [2]. In surgery, those new ideas could be a new surgical method, a new instrument or a procedure. However, if a new surgical process or instrument is introduced and used, many steps should be taken. 

Application of formal processes to include innovative surgical procedures into hospitals or health services has long been integrated into the healthcare system, while protecting the interests of patients, physicians and the organization [3]. Most countries have specific guidelines for assessing and introducing new surgical methods in hospitals and medical centers which are more or less similar to each other. The milestone of these standards hinges on the importance of the patient’s safety. Any new method should constitute no risk to patient’s health. Sometimes introducing new methods or developing new instruments, especially in the field of ocular surgery, might be hazardous to the patient can be potentially blinding procedure unless its safety has been already proven. 

Frequently the assessment as to whether a new surgical method should be introduced is between the desire to enhance knowledge and increase surgical experience against the potential risks of novel procedures. As a matter of fact, a substantial amount of sensory data is filtered through the eyes, they are our actually windows to the world hence the aim is to protect the visual system healthy against any risky procedure. Furthermore, the present and future costs of the new techniques and procedures should be estimated as precisely as possible. New methods must prove to be lower in cost than the older methods and both the morbidity and mortality rates should be demonstrably lower.

**Scope of the journal in surgical techniques**

Our journal intends to introduce new methods and instruments or convey the idea of creating them. A major difference between this journal and fiction is that the ideas should be evidence-based and must have been written based on scientific principles and logical procedures. 

Although the authors bear the responsibility for any datum they publish, we do not recommend using an idea or hypothesis in clinical setting only because they have been published in this journal. This journal is merely a pathway to more studies. It is absolutely necessary to run more laboratory and clinical studies and other research to show the reliability and feasibility of those findings. We recommend that researchers who are willing to publish new surgical methods or instruments point out the advantages and disadvantages of the discussed methods in a special section in their manuscript.

Another issue is training. That is, how to assess the amount of time needed for learning a new method, and if learning that method will be time-saving. The authors should clearly answer the questions that address the scope of training required for the instrument or new surgical techniques (e.g., how much content, what level of complexity)? Who is the audience of the new technique or an instrument (e.g., their experience, job requirements)? What resources are available (e.g., trainers, facilities, quick-reference guidelines)? Has the method been evaluated before? And how wide-ranging or complicated is the procedure? 

The other important subject is the ethical issues. It is important that a new method be evaluated for its ethical principles and whether there are any conflicts of interest. If a company has already started investing in a specific product, it should disclose its material interest to the journal. Researchers should also disclose how many patients the new ideas or devices have been tested upon and, more importantly, whether the animal studies had been performed beforehand. Furthermore, the researcher should declare his/her informed consent and obtain the ethics committee’s confirmation for a new technique or device. It is the patient’s right to choose and select his surgical methods based on their safety. The journal might ask for original confirmation of ethics committee. If a new method is introduced, the author should provide an explanation of which group of surgeons and with what level of surgical skills could use that method, and whether trainees would be able to perform them. If the author describes monitoring and the long term follow-up of the new technique, the manuscript would have a much greater chance of being published. 

**Spiritual ownership**

Spiritual ownership starts from the development of an idea. The registration of a new idea requires some tests that are both time-consuming and costly. Registry of the idea or technique in a journal such as ours gives the idea developer the opportunity to have his idea patented. As well, if he does not have the facilities to perform the necessary confirmations, the idea’s presence in the journal will allow others to do so.

The journal would publish papers even if the author does not have the opportunity to perform clinical researches. Since the researcher has generated the first spark for the idea, by publishing it, he can patent the idea or technique for himself. Having said that, we do declare that in legal terms, as patenting ideas and hypotheses has not become operational in many countries, we are unable to undertake the legal responsibility of the patent registration. The researcher should arrange for registering the patent for his idea, thought, instrument or medication by him/herself solely or with the collaboration of the university and scientific centers in his/her own country.

**Future directions**

In future issues we will continue to focus on original research and would also report important, timely information about innovation and ideas in ophthalmology and possible standards. However, ophthalmology and visual sciences manuscripts from perspectives such as ethics, ocular healthcare reform, and interactive comments from round table features are most welcome.

The scientific societies strongly oppose traditional methods and seek changes. Changes must be directed towards progress and address at least one of the problems facing humanity today. We thank those who make up this community: numerous individuals, authors and peer reviewers who have worked through the start-up process to ensure that our peer-review system is rigorous and sound. 

Finally, thanks to the editorial board members for their continuous guidance, trust, and support. 

We should endeavor to work together in order to create ideas to form a healthier and safer world for everybody!

## DISCLOSURE

The authors report no conflicts of interest in this work.
